# Evaluation of bond strength of various epoxy resin based sealers in oval shaped root canals

**DOI:** 10.1186/s12903-016-0301-1

**Published:** 2016-09-30

**Authors:** Fatih Cakici, Elif Bahar Cakici, Kadir Tolga Ceyhanli, Ersan Celik, Funda Fundaoglu Kucukekenci, Arif Onur Gunseren

**Affiliations:** 1 Department of Endodontics, Faculty of Dentistry, Ordu University, 52000 Ordu, Turkey; 2 Department of Endodontics, Faculty of Dentistry, Karadeniz technical University, Trabzon, Turkey; 3 Department of Prosthodontics, Faculty of Dentistry, Ordu University, Ordu, Turkey

**Keywords:** Bond strength, Epoxy resin-based sealers, Root canal filling, Ultrasound

## Abstract

**Background:**

The aim of this study was to evaluate the bond strength of AH plus, Acroseal, and Adseal to the root canal dentin.

**Methods:**

A total of 36 single-rooted, mandibular premolar teeth were used. Root canal shaping procedures were performed with ProTaper rotary instruments (Dentsply Maillefer) up to size F4. The prepared samples were then randomly assembled into 3 groups (*n* = 12). For each group, an ultrasonic tip (size 15, 0.02 taper) which was also coated with an epoxy resin based sealer and placed 2 mm shorter than the working length. The sealer was then activated for 10 s. A push-out test was used to measure the bond strength between the root canal dentine and the sealer. Kruskal-Wallis test to evaluate the push-out bond strength of epoxy based sealer (*P* = 0.05). The failure mode data were statistically analyzed using Pearson’s chi square test (*P* = 0.05).

**Results:**

Kruskal-Wallis test indicated that there were no statistically significant difference among the push out bond strength values of 3 mm (*p* = 0.123) and 6 mm (*P* = 0.057) for groups, there was statistically significant difference push out bond strength value of 9 mm (*P* = 0.032). Pearson’s chi square test showed statistically significant differences for the failure types among the groups.

**Conclusion:**

Various epoxy resin based sealers activated ultrasonically showed similar bond strength in oval shaped root canals. Apical sections for all groups have higher push out bond strength values than middle and coronal sections.

**Electronic supplementary material:**

The online version of this article (doi:10.1186/s12903-016-0301-1) contains supplementary material, which is available to authorized users.

## Background

The sealing of a root canal system completely prevents the colonization and reinfection of the oral pathogens in the root canal and periapical tissues; consequently, this procedure can provide a long-term successful endodontic treatment [1, 2]. Gutta-percha cannot adhere to the dentinal surface so sealer application is required. The activation of root canal sealers can possibly support their penetration inside dentinal tubules, providing an increase in the sealability and antibacterial effects [3]. Many researchers have investigated various irrigation techniques and ultrasonic systems to increase the push-out bond strength of sealers [4–6].

Epoxy resin-based sealers have excellent physical properties such as longer setting time, low solubility, high flow rate, low volumetric polymerization shrinkage, and interfacial adaptation and also are related to covalent bonds between epoxide rings and the exposed amino groups in the collagen network [7, 8].

Recently, Guimaraes et al. [9] showed that ultrasonic activation of an epoxy resin based sealer increased dentinal sealer penetration and decreased gaps. However, there are not enough sources in the literature for validating this data, so the effect of ultrasonic activation of the epoxy resin-based sealers on the push-out bond strength is unknown. Thus, the aim of this study was to evaluate the bond strength of AH plus (Dentsply Maillefer, Ballaigues, Switzerland), Acroseal (Septodont, Saint Maur des Fosses, France) and Adseal (Meta, Biomed, Cheongju, South Korea). The null hypothesis being tested was that there was no difference between the epoxy resin-based sealers (AH plus, Acroseal, Adseal) in terms of bond strength to root dentin.

## Methods

This study was approved by the Ethics Committee of Ordu University (2016/16). In our study, 36 single-rooted human mandibular premolar teeth which were extracted because of periodontal, prosthetic, and orthodontic reasons at the Department of Maxillofacial Surgery, Ordu University were used. Preoperative mesiodistal and buccolingual radiographs were taken of each root to confirm the canal anatomy. The teeth having more than one single root canal and apical foramen, root canal treatment, internal/external resorption, immature root apices, and caries/cracks/fractures on the root surfaces were excluded. The soft tissue and calculus on the root surfaces were mechanically removed by using a periodontal scaler and then the teeth were kept in distilled water at room temperature until they were used.

Only oval shaped root canals were used. To identify oval shaped canals, the minimum diameter of the root canal was measured mesio-distally, and the maximum diameter was measured bucco-lingually. Only teeth canals having buccolingual /mesiodistal ratios of more than 2 were used [10]. The size of the apical foramen was controlled by inserting a #10 stainless steel K-file (Dentsply Maillefer). Apical size greater than ISO size 10 was excluded from the study. The teeth were decoronated with a diamond disc under water cooling to obtain a standardized root length of 12 mm. Then, a #10 stainless steel K-file was inserted into the canal until its tip was just visible at the apical foramen and working length was determined as 1 mm short of this measurement. Root canal shaping procedures were performed with ProTaper rotary instruments (Dentsply Maillefer) up to the F4 (size 40, .06 taper) master apical file size. The root canals were irrigated with 2 ml 2.5 % NaOCl (ImidentMed, Konya, Turkey) after use of each file. At the end of the shaping, the root canals were irrigated with 5 ml of 17 % Ethylenediaminetetraacetic acid for 1 min and 5 ml of 2.5 % NaOCl for 1 min. The root canals were dried with paper points. Then the prepared samples were randomly assembled into 3 groups (*n* = 12) and filled as follows:

### AH plus with ultrasonic activation group

The circumference of the ultrasonic tip (size 15, 0.02 taper) was covered with the freshly mixed AH plus sealer attached to an ultrasonic device (NSK Varios 750; Nakanishi Inc., Tochigi, Japan). The tip was placed 2 mm shorter than the working length and the sealer was activated for 10 s. The root canals were filled with gutta-percha cones which were coated with a thin layer of the freshly mixed AH plus using cold lateral compaction technique.

### Acroseal with ultrasonic activation group

The circumference of the ultrasonic tip (size 15, 0.02 taper) was covered with the freshly mixed Acroseal sealer. The tip was placed 2 mm shorter than the working length and the sealer was activated for 10 s. The root canals were filled with gutta-percha cones which were coated with a thin layer of the freshly mixed Acroseal using cold lateral compaction technique.

### Adseal with ultrasonic activation group

The freshly mixed Adseal was applied around the circumference of the ultrasonic tip and then the tip was placed 2 mm shorter than the working length and the sealer was activated for 10 s. The root canals were filled with gutta-percha cones which were coated with a thin layer of the freshly mixed Adseal using cold lateral compaction technique.

After the filling procedures, mesio-distal and bucco-lingual radiographs were taken to confirm complete filling. Coronal cavities were filled with glass ionomer cement. The teeth were stored in an incubator at 37° C and 100 % humidity for 2 weeks to allow complete setting of the sealer.

All of the filled roots were separated perpendicular to their long axis using a precision saw (IsoMet 1000; Buehler, Lake Bluff, IL, USA) at a low speed under water cooling. Three slices were obtained from the each tooth at 3 mm, 6 mm, and 9 mm. The diameter of each hole from the apical and coronal aspects was measured under a stereomicroscope (Zeiss Stemi 2000C; Carl Zeiss; Jena; Germany) at 32× magnification. The push-out test was performed on the apical side of each section with a universal test machine (Lloyd Instruments, Fareham, UK) at a crosshead speed of 1 mm per minute; by applying a continuous load. Cylindrical pluggers, matching the diameter of each root parts, with the following diameters were used: 0.6 mm, 0.7 mm, and 0.8 mm. The diameter of the pluggers was approximately (at least) 80 % of the diameter of the canal. The maximum load applied to the filling material before failure was recorded in Newtons (N) and converted to mega Pascals (MPa) according to the following formula:

Push-out bond strength (MPa) = maximum load (N)/adhesion area of root filling (A) (mm^2^).

The adhesion area of the root canal filling was calculated using the following equation:

$\mathrm{A}=\left({\uppi \mathrm{r}}_1 + {\uppi \mathrm{r}}_2\right)\mathrm{X}\kern0.5em \mathrm{L},\mathrm{where}\kern0.5em \mathrm{L}=\sqrt[2]{{\left(\mathrm{r}1-\mathrm{r}2\right)}^2+h2}$, where r_1_ is the smaller radius, r_2_ is the larger radius of the canal diameter (mm), h represents the thickness of the root section (mm) and π is the constant 3.14.

After the test procedure, each section of the samples was visually examined under a stereomicroscope at 32× magnification to determine the type of failure. Three types of failure were categorized: adhesive failure (no sealer visible on dentine walls), cohesive failure (dentine walls totally covered with sealer) and mixed (a combination of cohesive and adhesive failure). The data of push-out bond strength of the root canal filling material to the root dentin were displayed as mean ± standard error of the mean (SEM), standard deviation and minimum-maximum values. The assumptions of data normality and homogeneity of variance, which are prerequisite for ANOVA, were tested with the Kolmogorov Smirnov and the Levene’s tests, respectively. When absence of normal distribution, nonparametric Kruskal-Wallis tests was performed and Mann-Whitney-*U* test was used for multiple comparisons. The frequency of failure type among the sections was analyzed with the Pearson chi-square test. The level of significance was set at *P* < .05. All calculations were performed with SPSS 23 (SPSS Inc, Chicago, IL) statistical software.

## Results

Table 1 shows the median, standard deviation and minimum - maximum push out bond strength values of groups. Kruskal Wallis test indicated that there were no statistically significant differences among the sealer groups for apical (*p* = 0.123) and middle sections (*P* = 0.057), there was statistically significant difference among the groups for coronal sections (*P* = 0.032) and Mann Whitney *U* test indicated that the difference was between Adseal and AH plus for coronal section (*P* = 0.009).

**Table 1:** Median and range push out bond strength value of sealers

Sealers	Sections	Number	Median	Std. Dev.	Min-Max
Adseal	apical	12	1.34 Aa	1.057	0.621-4.210
	middle	12	0.425Ab	0.579	0.001-1.948
	coronal	12	0.000Bb	0.938	0.001-2.816
Acroseal	apical	12	2.19Aa	1.223	0.688-4.180
	middle	12	0.905Ab	0.317	0.098-1.220
	coronal	12	0.87Bb	0.739	0.001-1.971
Ah Plus	apical	12	2.72Aa	2.330	0.305-7.342
	middle	12	1.005Ab	0.626	0.208-2.099
	coronal	12	2.21Bb	4.510	0.150-12.28

median of sections that do not share a capital letter in the same sealers are significantly different

median of sealers that do not share a small letter in the same thickness are significantly different

According to the sealer types, there were statistically significant differences among the sections of Adseal and Acroseal, the differences were between both apical-middle and apical-coronal sections (*P* < 0.05) and there was no statistically significant difference between middle- coronal sections for both sealers (*P* > 0.05). For AH plus, there was a statistically significant difference between apical-coronal sections (*P* < 0.05) and there were no statistically significant differences for both apical-middle and middle-coronal sections (*P* > 0.05).

The Pearson chi-square test indicated that there were statistically significant relationship for frequency of failure types in apical middle and coronal sections and there were no statistically significant difference among the gropus interms of failure types in Adseal, Acroseal and AH Plus (Table 2).

**Table 2:** Frequency analysis and Chi-square test results for failure type

	Failure type	Adseal		Acroseal		Ah plus		*P*-Value
		Frequency	%	Frequency	%	Frequency	%	
apical	ADHESIVE	1	2.78	1	2.78	2	5.56	0.000**
	COHESIVE	5	13.89	3	8.33	3	8.33	
	MIX	6	16.67	8	22.22	7	19.46	
middle	ADHESIVE	1	2.78	1	2.78	1	2.78	0.000^**^
	COHESIVE	8	22.22	2	5.56	6	16.67	
	MIX	3	8.33	9	25.02	5	13.9	
coronal	ADHESIVE	0	0	2	2.78	2	5.56	0.000^**^
	COHESIVE	3	8.33	3	8,33	4	11.12	
	MIX	9	25.02	7	19,46	6	16.67	
*P*-Value		0.173^NS^		0.910^NS^		0.771^NS^		

**Statistically significant (*p* < 0.01); ^NS^Not statistically significant (*p* > 0.05)

## Discussion

The effect of ultrasonic activation of a sealer on bond strength was investigated in this study. The null hypothesis was that there would be no difference among the groups in terms of the push-out bond strength. According to results of the present study, there was no statistically significant difference among the push-out bond strength values of epoxy resin based sealers in the apical and middle sections. Thus, the null hypothesis was partially accepted. The results of present study show that the failure types among the sections for groups were found significant statistically.

The penetration of the sealer into the dentine tubules of the root canal changes according to the type of sealer, the irrigation systems and solutions [4, 6, 9]. Ultrasonic devices are commonly used to increase the efficacy of the irrigating solutions [5, 11].

Duarte et al. [12] evaluated the effect of ultrasonic activation of calcium hydroxide pastes on pH and calcium release in simulated external root resorptions. They found a higher pH level when the calcium hydroxide paste was activated with ultrasound. They reported that this outcome was possibly due to the positive effect of ultrasonic activation on the penetration of calcium hydroxide particles inside of the dentinal tubules. In our study, irrigation solution and irrigation systems were standardized. Conversely, the sealer was activated ultrasonically while the root canals were being filled.

Marciano et al. [13] reported that there were no statistical differences in terms of adaptation, percentage of voids, solubility and flow among the epoxy resin based sealers (AH Plus, Adseal and Acroseal). More recently, another study by Guimaraes et al. [9] evaluated the effects of ultrasonic activation on the filling quality (intratubular sealer penetration, interfacial adaptation and presence of voids) of four epoxy resin based sealers and concluded that ultrasonic activation of epoxy resin based sealer promoted greater dentinal sealer penetration and reduced the presence of gaps. Moreover, they found a significant increase in penetration for the AH Plus, Acroseal, and Sealer 26 at the 4-mm level, and the AH Plus and Sealer 26 at the 6-mm level when the sealers were activated ultrasonically. The effect of ultrasonic activation on the push-out bond strength of epoxy resin based sealers was evaluated in the present study. To the best our knowledge, there is no available data about this issue in the literature. Consequently, the findings of our study cannot be directly compared with the findings of the aforementioned study. Current study results show that apical section has the highest push out bond strength values than middle and coronal sections for epoxy resin based sealers. Because the taper of master apical file is 0.06 and the taper of ultrasonic tip is 0.02, in apical section the distance between dentin wall and ultrasonic tip is shorter than middle and coronal sections of root. Therefore the ultrasonic activation may be more effective in apical section.

## Conclusion

Within the limitations of this study, various epoxy resin based sealers activated ultrasonically showed similar bond strength in oval shaped root canals. Apical sections for all groups have higher push out bond strength values than middle and coronal sections.

## Additional file

Additional file 1:
**Table S1.** Failure type and push out bond strength value of Acroseal. **Table S2.** Failure type and push out bond strength value of AH plus. **Table S3.** Failure type and push out bond strength value of Adseal. (DOCX 15 kb)
