# Supplementary material for: Evaluation of bond strength of various epoxy resin based sealers in oval shaped root canals
Source: BMC Oral Health. 2016 Sep 30;16:106. doi: 10.1186/s12903-016-0301-1 (PMC5045642; doi:10.1186/s12903-016-0301-1)
Supplement: Additional file 1: — Table S1. Failure type and push out bond strength value of Acroseal. Table S2. Failure type and push out bond strength value of AH plus. Table S3. Failure type and push out bond strength value of Adseal. (DOCX 15 kb) [file 12903_2016_301_MOESM1_ESM.docx]

| ACROSEAL 3MM | FAILURE TYPE | ACROSEAL 6MM | FAILURE TYPE | ACROSEAL 9 MM | FAILURE TYPE |
| --- | --- | --- | --- | --- | --- |
| 0,79 | MIXT | 1,22 | MIXT | 0,00 | MIXT |
| 2,74 | MIXT | 0,64 | COHESIVE | 0,00 | MIXT |
| 2,72 | MIXT | 0,84 | MIXT | 1,59 | ADHESIVE |
| 3,41 | COHESIVE | 0,10 | MIXT | 0,90 | MIXT |
| 0,69 | MIXT | 1,10 | MIXT | 1,09 | MIXT |
| 0,86 | ADHESIVE | 1,05 | MIXT | 1,88 | MIXT |
| 1,23 | COHESIVE | 1,19 | MIXT | 0,46 | ADHESIVE |
| 3,46 | MIXT | 0,91 | MIXT | 1,03 | MIXT |
| 4,18 | MIXT | 0,90 | COHESIVE | 0,00 | COHESIVE |
| 0,96 | MIXT | 1,12 | ADHESIVE | 0,00 | COHESIVE |
| 1,71 | COHESIVE | 0,86 | MIXT | 1,97 | MIXT |
| 2,67 | MIXT | 0,59 | MIXT | 0,84 | COHESIVE |

Table 1. Failure type and push out bond strength value of Acroseal

| AH plus 3MM | FAILURE TYPE | AH plus 6MM | FAILURE TYPE | AH plus 9 MM | FAILURE TYPE |
| --- | --- | --- | --- | --- | --- |
| 1,57 | mixt | 0,21 | Mixt | 11,01 | mixt |
| 5,09 | adhesive | 0,34 | Mixt | 0,34 | cohesive |
| 5,40 | mixt | 0,94 | Adhesive | 6,78 | adhesive |
| 7,25 | mixt | 1,67 | Cohesive | 3,28 | mixt |
| 4,51 | mixt | 1,27 | Cohesive | 0,37 | adhesive |
| 7,34 | cohesive | 2,10 | Cohesive | 12,28 | mixt |
| 1,60 | mixt | 0,54 | Mixt | 8,73 | mixt |
| 2,41 | adhesive | 1,07 | Cohesive | 0,29 | cohesive |
| 0,31 | mixt | 0,26 | Cohesive | 2,27 | mixt |
| 3,03 | mixt | 0,47 | Mixt | 2,15 | cohesive |
| 1,81 | cohesive | 1,62 | Cohesive | 0,24 | cohesive |
| 2,09 | cohesive | 1,29 | Mixt | 0,15 | mixt |

Table 2. Failure type and push out bond strength value of AH plus

| ADSEAL 3MM | FAILURE TYPE | ADSEAL 6MM | FAILURE TYPE | ADSEAL 9 MM | FAILURE TYPE |
| --- | --- | --- | --- | --- | --- |
| 1,34 | MIXT | 1,03 | COHESIVE | 0,00 | COHESIVE |
| 1,21 | COHESIVE | 1,95 | COHESIVE | 0,00 | MIXT |
| 2,47 | MIXT | 0,22 | COHESIVE | 1,22 | MIXT |
| 1,23 | MIXT | 0,10 | MIXT | 1,65 | COHESIVE |
| 2,47 | COHESIVE | 0,77 | ADHESIVE | 2,82 | COHESIVE |
| 1,14 | COHESIVE | 0,74 | COHESIVE | 0,00 | MIXT |
| 4,21 | ADHESIVE | 0,00 | MIXT | 0,00 | MIXT |
| 1,34 | COHESIVE | 0,08 | MIXT | 1,43 | MIXT |
| 3,05 | MIXT | 0,15 | COHESIVE | 0,00 | MIXT |
| 0,62 | COHESIVE | 0,89 | COHESIVE | 0,00 | MIXT |
| 0,85 | MIXT | 0,00 | COHESIVE | 0,97 | MIXT |
| 2,30 | MIXT | 0,63 | COHESIVE | 0,00 | MIXT |

Table 3. Failure type and push out bond strength value of Adseal
